# Identification and assessment of virulence of a natural reassortant of infectious bursal disease virus

**DOI:** 10.1186/s13567-018-0586-y

**Published:** 2018-09-12

**Authors:** Anna Pikuła, Anna Lisowska, Agnieszka Jasik, Krzysztof Śmietanka

**Affiliations:** 1 Department of Poultry Diseases, National Veterinary Research Institute, Al. Partyzantow 57, 24-100 Pulawy, Poland; 2 Department of Pathology, National Veterinary Research Institute, Al. Partyzantow 57, 24-100 Pulawy, Poland

## Abstract

Infectious bursal disease virus (IBDV) is one of the most important immunosuppressive viral agents in poultry production. Prophylactic vaccinations of chicken flocks are the primary tool for disease control. Widely used immunoprophylaxis can, however, provide high pressure which contributes to the genetic diversification of circulating viruses, e.g. through reassortment of genome segments. We report the genetic and phenotypic characterization of a field reassortant IBDV (designated as Bpop/03) that acquired segment A from very virulent IBDV and segment B from classical attenuated D78-like IBDV. Despite the mosaic genetic make-up, the virus caused high mortality (80%) in experimentally infected SPF chickens and induced lesions typical of the acute form of IBD. The in vivo study results are in contrast with the foregoing experimental investigations in which the natural reassortants exhibited an intermediate pathotype, and underline the complex nature of IBDV virulence.

## Introduction

Infectious bursal disease virus (IBDV) is a double-stranded RNA virus which belongs to the *Avibirnavirus* genus, *Birnaviridae* family. The virus possesses a bi-segmented genome (segments A and B) within a non-enveloped icosahedral capsid and is the causative agent of an immunosuppressive disease of young chickens [1]. It is commonplace in nature that viruses with segmented genomes may exchange genetic material if they infect one cell at the same time. Natural reassortants of IBDV have already been reported from Asia [2, 3], South America [4], North America [5], Africa [6, 7] and Europe [8].

The genome of IBDV encodes for 5 viral proteins. VP2 is the major structural viral protein that builds the viral capsid [9] and contains an antigenic domain responsible for inducing secretion of neutralization antibodies [10]. The IBDV encodes its own RNA-dependent RNA polymerase designated VP1 that is responsible for the genome replication, transcription [11] and probably translation of virus protein [12]. The VP2, VP1 and another structural protein VP3 together play a crucial role in the morphogenesis and encapsidation of the viral genome [13]. In turn, the VP4 protein is a viral protease [14] that effects post-translational cleavage of the polyprotein (VP2–VP4–VP3) and takes part in the maturing of the VP2 peptide [15]. VP5 is a non-structural protein that cooperates in releasing of virus progeny from infected cells both through the non-lytic mechanism during the early phase of infection [16] and the activation of cell apoptosis in later stages by interacting with mitochondrial ionic channels (VDAC2) [17].

IBDV serotype 1 is pathogenic to chickens but the virulence among this group can vary. The very virulent IBDV (vvIBDV) are the most pathogenic and can cause mortality up to 100% in SPF chickens. Classical and US variant strains are less virulent but they are immunosuppressive. The attenuated strains are generated by passaging different virus pathotypes on cell culture or SPF chicken embryos resulting in loss of pathogenic capacity [1]. Such strains are used in commercial live vaccines.

IBD has been reported in Poland since the end of the 1970s when the first epidemic caused by classical strains occurred. At the end of 1991, the first cases of acute IBDV were diagnosed. Infections with very virulent IBDV have caused economic losses due to high mortality rates. Afterwards, new immunoprophylaxis programmes were implemented which reduced the number of vvIBDV infections, but nevertheless IBD was still affecting polish poultry production [18]. Some of the Polish classical and very virulent strains have been antigenically and molecularly characterized [19] but since then there has been a lack of information about the recent field IBD situation in this part of Europe.

The aim of this study was a genetic and pathotypic characterization of a reassortant IBD virus detected in Poland.

## Materials and methods

### Samples

Samples were collected in 2003 from vaccinated broiler chickens (Nobilis D78, MSD Netherlands) with signs of immunosuppressive disorder. Ten bursa samples were collected for laboratory examinations. The tissue samples were homogenised in phosphate buffered saline (4 mL PBS per gram tissue) and centrifuged at 4 °C 3500* g* for 15 min. The fluid obtained was collected and stored (below −65 °C) for further investigations.

### Virus isolation

The virus isolation was conducted on 10-day-old SPF embryonated eggs using the chorioallantoic membrane route (CAM) [20]. The fluid was filtered through a 0.45 µm membrane before inoculation. After 7-day incubation the embryos and chorioallantoic membrane were harvested, homogenised and stored below −65 °C.

### Detection and identification of IBDV

The total RNA was extracted using an RNeasy Mini Kit (Qiagen, Germany) according to the manufacturer’s instructions. The presence of the IBD virus in bursal and embryo-derived samples was confirmed with adherence to a previously published rRT-PCR protocol [21]. Identification of the IBDV genotype was performed according to a previously described RT-PCR aimed at the hypervariable domain of VP2 peptide [22]. The partial sequence of VP1 peptide was amplified with primers designed to the nucleotide sequence of D6948 strain (GenBank Accession number AF240687, Table 1) which are complementary to the 5′ two-thirds of segment B. The RT-PCR reaction was performed in a 25 μL volume of reaction mixture using the one-step RT-PCR kit (Qiagen, Germany). The reverse transcription step was conducted at 50 °C for 30 min and PCR was performed under the following conditions: initial denaturation at 95 °C for 15 min, followed by 35 cycles of amplification (denaturation 94 °C for 40 s, annealing at 58 °C for 1 min and elongation at 72 °C for 1 min). Final extension was conducted at 72 °C for 7 min. The PCR products obtained were sequenced in both directions by a commercial service (Genomed, Poland).

**Table 1:** **Primer sequences used in this study for the amplification of a partial sequence of VP1 gene**

Primer	Sequence (5′→ 3′)	Nucleotide position according to D6948 strain (AF240687)	Size of amplicon (bp)
SB1F	GTGGAAGAACTCCTGATCCCTAA	193–215	943
SB1R	GATGGAGCTGACCATATGTT	1117–1136	
SB2F	AACAAGAAGAAGCTGCTCAGC	1006–1026	854
SB2R	GATCCCAAGATCTTTGCTGTA	1840–1860	

### Nucleotide sequence and analysis

Molecular characterization and phylogenetic analysis was performed in MEGA 6 [23]. The nucleotide and amino acid sequences were aligned with the ClustalW algorithm. Phylogenetic trees were constructed using the neighbour-joining method with 1000 replicates. A bootstrap value over 70 was considered reliable for the tree topology.

### Full-length sequencing of genomic segments by high-throughput sequencing

The IBDV virus suspension was centrifuged at 3500 *g* for 5 min at 4 °C and filtered through a 0.45 µm membrane (Sartorius, Germany). For nuclease treatment 180 µL of virus supernatant was incubated for 30 min at 37 °C with the addition of 40 units of DNase I (Turbo DNase, Invitrogen, Lithuania) and 20 units of ribonuclease (RNase One, Promega, USA). Afterwards, TRI Reagent extraction of total RNA was conducted using a Direct-zol RNA MiniPrep Kit (Zymo Research, USA) according to the supplier’s instructions. The eluted RNA was subsequently subjected to reverse transcription with the use of the SuperScript IV First-Strand Synthesis System (Invitrogen, Lithuania) and random primers.

The libraries were created using the Ilumina Nextera XT DNA Library Preparation Kit according to the manufacturer’s protocol and afterwards were sequenced using the MiSeq System (Ilumina, USA) with 2 × 300 paired-end reads. The sequences obtained were first mapped using BLAST analysis with the NCBI database in order to remove host-derived sequences (*Gallus gallus*). The remaining sequences were de novo assembled into contigs using the SPAdes genome assembler (version 3.11.1) [24].

### Pathogenicity in chickens

Animal experiments were carried out in accordance with the requirements and authorization of the local ethical commission (Permit no. 87/2012). The D78 (Nobilis, Intervet, Netherlands) and 75/11 were used as a reference for attenuated and typical very virulent strains. Before the animal trial, the 75/11 strain was characterised genetically (partial sequence of VP2: KX759595 and VP1: KX759549) and pathotypically (60% mortality in 5-week old SPF chickens). Based on the OIE standards [20], it was classified as vvIBDV (data not published). Forty five-week-old SPF-layers (White Leghorn, Valo BioMedia, Germany) were divided into 4 groups (10 birds/group). Birds from groups 1 and 2 were intraocularly inoculated with 10^4^ EID_50_/0.1 mL of Bpop/03 virus suspension (the reassortant strain) and 75/11 virus (vvIBDV strain), respectively. Chickens from group 3 were inoculated with 1 dose of vaccine D78 strain (attenuated IBDV control) and the 4th group was given saline buffer and served as a normal control group. Birds were housed in HEPA-filtered isolators (Montair Andersen B.V., Holland) for 10 days. All chickens were observed daily. All dead or euthanized birds were examined at necropsy and the bursae of Fabricius (BF) were taken for further analysis (histopathology, molecular characterisation, and calculation of bursa to bodyweight (B-BW) index).

### Histopathology

Half of each bursa was fixed in 10% phosphate-buffered formalin. In further proceedings the tissues were embedded in paraffin, sectioned and stained using haematoxylin and eosin. The preparations were microscopically examined and evaluated for histopathological abnormalities according to the criteria described by Jackwood et al. [5].

### Statistical analysis

The B-BW indexes obtained from each experimental group versus the control group were compared by means of the Mann–Whitney U test, using the social science statistics on-line calculator [25]. To assess if differences in mortality between groups of chickens infected with typical and reassortant IBDV are statistically significant, the Chi square (χ^2^) test was used. The results with *P* < 0.05 were recognised as statistically significant.

### Identification of concomitant pathogens

In order to reveal the presence of any concomitant pathogens, next-generation sequencing of extracted total DNA and RNA in Bpop/03 samples were performed. The total DNA was purified using a QIAamp DNA Mini kit (Qiagen, Germany) in accordance with the manufacturer’s instructions. The purification of total RNA was described in the previous subsection.

## Results

### Detection of IBDV

The rRT-PCR reaction confirmed the presence of IBDV in the examined flock and the presence of virus in inoculated SPF embryos. Further identification with conventional RT-PCR, sequencing and BLAST searches of partial sequences of both segments revealed that the analysed VP2 gene sequence has high nucleotide identity to the very virulent D6948 strain (Accession Number AF240687) and VP1 gene sequence to the cell culture–adapted attenuated 903/78 strain (JQ411013).

### Sequence analysis

The nearly-complete consensus genome sequence of the Bpop/03 isolate obtained by NGS technology was submitted to GenBank (Accession Numbers: segment A—MH545934, segment B—MH545935). The sequences were aligned using the BLAST tool with the NCBI database. Most of the available IBDV sequences were retrieved and used for further molecular analysis (listed in Table 2). The alignment of both segment sequences confirmed the reassortant nature of the Bpop/03 isolate. The comparison of segment A and B nucleotide sequences revealed 99.1% similarity to the European vvIBDV strain D6948 and 99.3% similarity to the attenuated strain 903/78.

**Table 2:** **List of IBDV’s used in phylogenetic analysis**

IBDV strain	Origin	Pathotype/serotype	Accession number	
			Segment A	Segment B
02015.1	Venezuela	Reassortant	AJ879932	AJ880090
100056	France	Reassortant	KU2345528	KU2345529
150124/1.1	Algeria	Reassortant	MF969105	MF969106
150144/5.1	Algeria	Reassortant	MF969115	MF969116
23/82	UK	Serotype 2	AF362773	AF362774
88180	Ivory Coast	Very virulent	AM111353	AM111354
89163	France	Very virulent	HG974563	HG974564
903/78	Hungary	Attenuated	AF362773	JQ411013
9109	USA	Classical	AY462027	AY459321
94432	France	Very virulent	AM167550	AM167551
BD 3/99	Bangladesh	Very virulent	AF362770	AF362776
CA-D495	USA	Reassortant	JF907703	JF907704
CA-K785	USA	Reassortant	JF907702	JF907705
Cro-Ig/02	Croatia	Very virulent	EU184685	EU184686
Cu-1	Germany	Attenuated	D00867	AF362775
D6948	Netherlands	Very virulent	AF240686	AF240687
D78	Nobilis	Attenuated	AF499929	EU162090
Edgar	USA	Classical	AY462026	AY918949
Faragher 52/70	UK	Classical	HG974565	HG974566
GLS	USA	Variant	AY368653	AY368654
Gt	China	Attenuated	DQ403248	DQ403249
Gx	China	Reassortant	AY444873	AY705393
GX-NN-L	China	Reassortant	JX134485	JX134486
Harbin-1	China	Reassortant	EF517528	EF517529
HBDY-1	China	Classical	KX592158	KX592159
HK46	China	Very virulent	AF092943	AF092944
HLJ-0504	China	Reassortant	GQ451330	GQ451331
HN	China	Reasortant	KC109816	KC109815
HuB-1	China	Very virulent	KF589805	GQ449693
HuN11	China	Reassortant	LM651367	LM651368
IBD13HeB01	China	Reassortant	KP676467	KP676468
Irwin Moulthrop	USA	Classical	AY029166	AY029165
K669	USA	Reassortant	JN585293	JN411134
ks	Israel	Very virulent	DQ927042	DQ927043
KZC-104	Zambia	Reassortant	AB368968	AB368969
Lukert	USA	Classical	AY918948	AY918947
NB	China	Very virulent	EU595667	EU595673
OH	USA	Serotype 2	–	U20950
OKYM	Japan	Very virulent	D49706	D49707
PA/00924/14	USA	Serotype 2	KP642112	KP642111
SD10LY01	China	Very virulent	KF569803	KF569804
SH95	China	Reassortant	AY134874	AY134875
SH99	China	Very virulent	LM651365	LM651366
T09	Nigeria	Very virulent	AY099456	AY099457
Tasik	Indonesia	Very virulent	AF322444	AF322445
TL2004	China	Reasortant	DQ088175	DQ118374
UK661	UK	Very virulent	AJ318896	AJ318897
UPM04/190	Malaysia	Very virulent	KU958716	KU958717
Variant E	USA	Variant	AF133904	AF133905
Winterfield 2512	Cevac	Attenuated	–	AF083092
ZJ2000	China	Reassortant	AF321056	DQ166818

The deduced amino acid sequence of segment A exhibited a profile typical for very virulent strains with the presence of four specific alterations–one each in the VP5 (F30) and VP2 proteins (L324) and two in VP3 (M778, I856) (Table 3). The first changed residue is located within the VP5 N-terminal domain that is responsible for the interaction with voltage-dependent anion channel 2 (VDAC2) [17] but without further investigation it is difficult to anticipate its impact on the activation of cell apoptosis process. However, the presence of leucine residue at position 324 leads to a loss of IBDV binding capacity with Mab 8 [26]. Finally, the role of the two changes within VP3 protein in IBDV virulence is unknown. The VP1 protein sequence comprised amino acids characteristic for attenuated strains with only one change at position 24 (A → V) and there is no information about the function of this region of VP1 in viral replication.

**Table 3:**
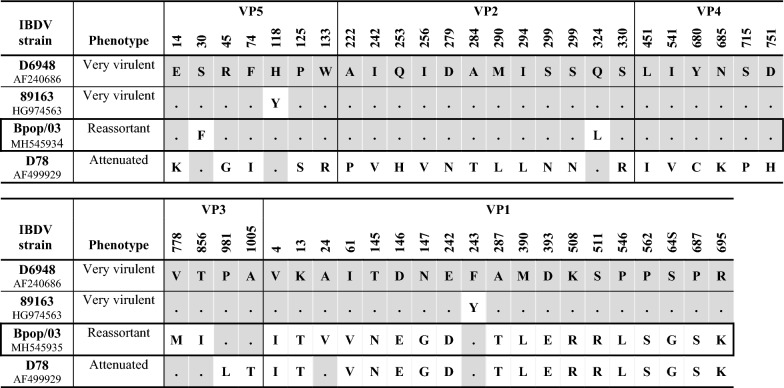
**Amino acid differences between typical vvIBDV (D6948, 89163), attenuated strain (D78) and Polish reassortant strain (Bpop/03)**

The deduced amino acid sequence of D6948 was used as a reference. Amino acid residues identical to reference strain were indicated by dots (.).

**Figure 1:**
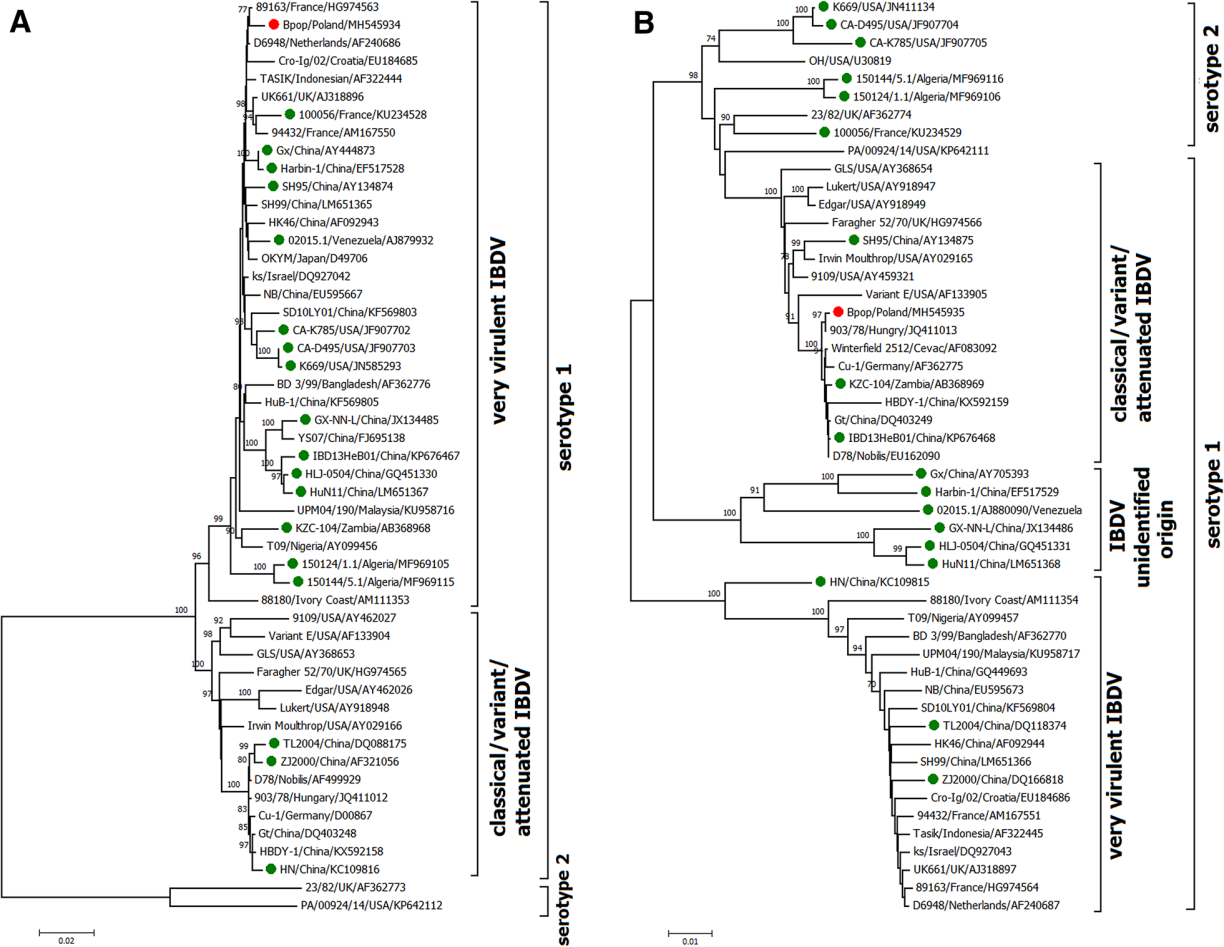
**Phylogenetic tree deduced from the full-length genome segment A (A) and B (B) of representative IBDV strains using neighbour joining methods (1000 replicates).** Red dot—polish reassortant strain, green dot—earlier identified reassortant strain.

The phylogenetic analysis also confirmed the different origins of both segment sequences. Based on the sequences of segment A, the 51 representatives of different geno- and serotypes of IBDV were clustered into three distinct branches designated as (i) serotype 2, (ii) very virulent and (iii) classical, variant and attenuated IBDV (Figure 1A). The Bpop/03 was found to be closely related to typical vvIBDV strains originating in Europe (D6948, 89 163 and UK661) and Asia (TASIK, Gx and OKYM). However the phylogenetic tree of segment B revealed that Bpop/03 formed a cluster with classical, variant and attenuated strains. Moreover the studied isolate was clustered on one branch with attenuated strains such as Winterfield 2512, Cu-1 and D78 (100% bootstrap).

### Pathogenicity

**Figure 2:**
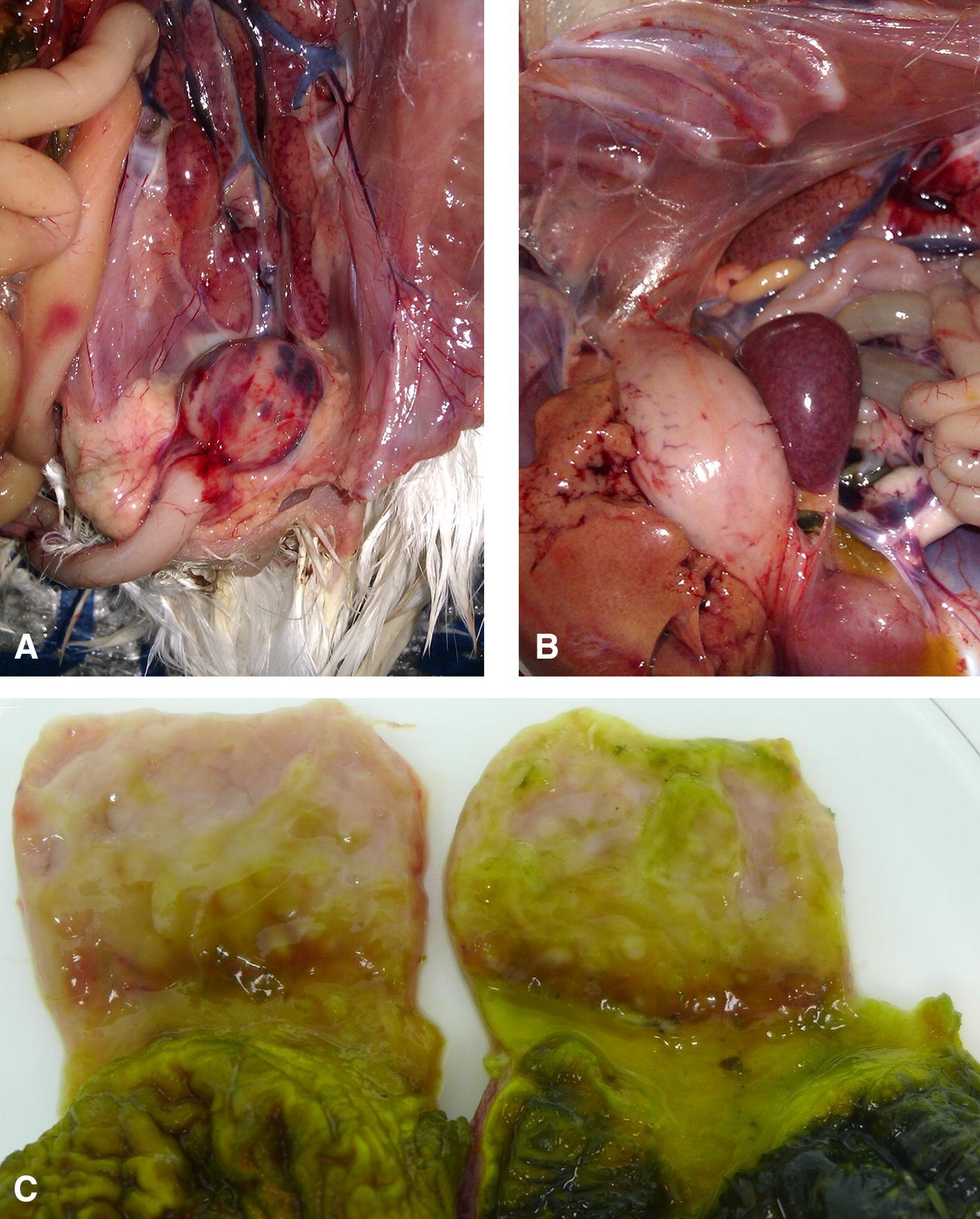
**Macroscopic changes observed at necropsy in chickens infected with reassortant strain (Bpop/03). A** Bursa of Fabricius and kidneys; **B** spleen; **C** proventriculus.

The infection of SPF chickens revealed the high pathogenicity of the Bpop/03 strain: 8 out of 10 SPF chickens died between 3 and 9 days post-infection (dpi). From the second day onwards, typical signs of IBD were observed, including ruffled feathers, depression and watery diarrhea. At necropsy, ecchymotic haemorrhages in the enlarged bursa of Fabricius, breast and thigh muscles and at the mucosal lining of the proventriculus, swollen spleens with punctate clusters of lymphoid tissue and swollen kidneys with visible tubule structure were observed (Figure 2). The bursae of chickens that died between 3 and 6 dpi were enlarged but on the 9th and 10th dpi the organ exhibited severe atrophy. Mortality in the control group inoculated with the reference very virulent IBDV reached 60% and chickens exhibited macroscopic lesions similar to those observed for the Bpop/03 strain. The differences in mortality rates as well as in B-BW ratios at days 9 and 10 post-infection between groups of chickens infected with Bpop/03 and 75/11 IBDV were not statistically significant. In contrast, B-BW indexes of both of these groups were found to be statistically significantly lower than those of the normal and D78 control groups (*P* < 0.05). No clinical symptoms or gross bursal lesions were observed in the D78 and normal control groups. Detailed results of the pathogenicity experiment conducted are presented in Table 4. Moreover the de novo next-generation sequencing showed the absence of any concomitant bacteria or virus in the investigated samples.

**Table 4:** **Results of in vivo IBDV virulence to SPF chickens**

Group no.	IBDV strain	Mortality (%)	B-BW index $\bar{x}$ ± SD		Histopathologic lesion scores^c^
			3–5 dpi	10 dpi	
1	Bpop/03 reassortant	8/10 (80)	4.53 ± 1.97 *n* = 6	1.43 ± 0.61^b^ *n* = 4	4, 4, 4, 4, 4, 4, 4, 4, 4, 4
2	75/11 vvIBDV control	6/10 (60)	5.73 ± 1.77 *n* = 6	1.45 ± 0.21 *n* = 4	4, 4, 4, 4, 4, 4, 2, 4, 4, 4
3	D78 attenuated IBDV control	0/10 (0)	nd	3.45 ± 1.09 *n* = 10	1, 2, 2, 2, 2, 2, 2, 1, 2, 2
4	Normal control	0/10 (0)	4.70 ± 0.19^a^ *n* = 5	5.44 ± 0.29 *n* = 5	1, 0, 0, 0, 0, 0, 0, 1, 0, 0

^a^From chickens euthanized at 3 dpi.

^b^From chickens dead at 9 dpi or euthanized at 10 dpi.

^c^According to Jackwood et al. [5], $\bar{x}$, arithmetic mean; SD: standard deviation; nd: not determined.

### Histopathology

**Figure 3:**
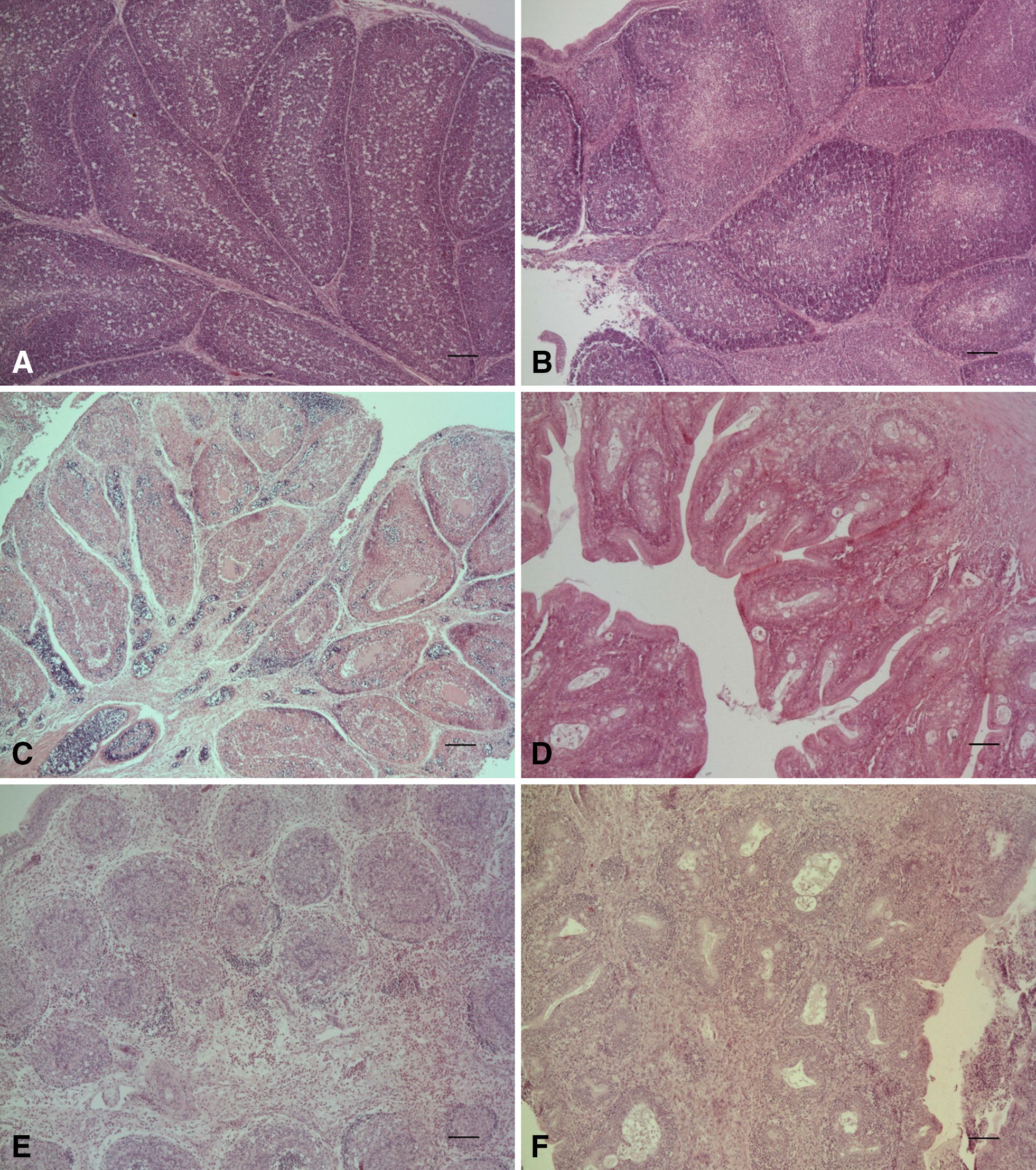
**Differences in the severity of bursa of Fabricius macroscopic lesions in examined groups. A** Normal group, **B** positive classic IBDV (D78) control group, **C** very virulent (75/11) control group at 3 dpi, **D** same group at 10 dpi, **E** Reassortant (Bpop/03) group at 3 dpi, and **F** same group at 10 dpi. The magnification is represented by the black band (×50).

Histopathological examination of the collected bursae of Fabricius from groups 1–3 indicated differences in the severity of microscopic lesions. The group inoculated with the vaccine D78 strain mainly caused low and moderate depletion of lymphatic follicles and hyperplasia of the *tunica muscularis* (Figure 3B). Bursa tissues microscopic alterations in groups challenged with Bpop/03 and 75/11 had a similar intensity. In birds found dead in the early phase of infection the lesions were mainly associated with the infiltration of inflammatory cells (heterophils and macrophages) in the *tunica muscularis* and interstitial connective tissue and depletion or necrosis of medullary and cortical regions of the lymphatic follicles due to the degeneration of lymphoid cells (Figures 3C and E). On the contrary, the bursal tissue collected from euthanized birds was characterized by the absence of lymphoid follicles due to necrosis, atrophy or fibrosis (Figures 3D and F).

In the microscopic examination of the mock-inoculated control the normal histological structure of the BF was recorded (Figure 3A) and only two birds showed a slight loss of lymphoid cells in the medulla of the lymphatic follicles.

## Discussion

Reassortment is one of the mechanisms of genetic diversity characteristic of viruses with segmented genomes. To emphasize the role of reassortment in virus genetic variability it is worth bringing up the example of influenza A virus, which each year brings new epidemiological threats to humans and animals [27]. The first IBDV reassortants from Venezuela were reported over a dozen years ago [4], but since then more such reassortant viruses have been identified all over the world. The results of investigations mostly revealed the exchange of genome segments between different pathotypes representing viruses of serotype 1 [1, 2, 6], but inter-serotype reassortants were also identified [5, 8].

The virulence of IBDV is a complex mechanism attributed to different components of the viral genome. Several mosaic IBDV constructed in vitro revealed the contribution of both segments A and B to virulence [28–31]. So far, most of the natural reassortant IBDV reported have exhibited intermediate virulence between very virulent and classical pathotype strains [4, 32].

To our knowledge, this study is the first report on the detection of new reassortant infectious bursal disease virus in Eastern Europe. Despite shuffling of genomic segments originating from two parental strains with distinct pathotypes (very virulent and classical cell-culture adapted), the new IBDV strain has inherited high virulence and represents a very virulent phenotype based on the established in vivo criteria [20]. The Bpop/03 IBDV strain caused 80% mortality in SPF chickens (indicative of high virulence), the birds had typical signs of IBD and post-mortem examinations revealed macro- and microscopic abnormalities in multiple organs including extensive lesions in the bursa of Fabricius. The presence of concomitant pathogens was rejected in the face of results from de novo high-throughput sequencing. Moreover, high-throughput sequencing did not provide evidence of mixed infection with other IBDV and therefore we assume that the investigated virus is a natural very virulent/attenuated reassortant that has retained high virulence. New IBDV strains that have preserved their pathogenicity despite the reassortment have been recently described [3, 7]. Li et al. [3] associated the preserved pathogenicity of the Chinese HLJ0504 strain with its novel segment B with unknown origin. Another two reassortants (150124 and 150244) from Algeria also exhibited comparable virulence to a very virulent reference strain (89163). In this case, the authors attributed the phenotype to the amino acid changes in the VP1 protein, but the fact is that both strains also possess several alterations of the VP5 protein that could modify its function. This part of the IBDV genome is quite conserved and plays a major role in IBDV virulence [33]. Sequence analysis of segment A of Bpop/03 revealed the presence of four unique residues within VP5 (F30), VP3 (M778, I856) and VP1 (V24). Because the molecular markers of IBDV virulence have not been defined, more investigations using reverse genetics are needed to explore the role of this mutation.

Despite the high nucleotide homology to the D78 strain (99.1%), it is unlikely that segment B of the reassortant IBDV was derived from a commercial vaccine seed because the genetic changes are too extensive. Probably the vaccine strain had been circulating in the farm environment for a longer period before it reassorted with the very virulent field strain.

In conclusion, a new natural reassortant of very virulent IBDV was identified in Poland. The virus retained its high virulence despite the acquisition of a segment B from the attenuated IBDV strain. This finding shows that the mechanism of IBDV virulence is intricate and merits further investigations.

## Abbreviations

B-BW: bursa to bodyweight index
BF: bursae of fabricius
BLAST: basic local alignment search tool
CAM: chorioallantoic membrane
EID_50_: mean embryo infective dose
HEPA: high efficiency particulate air filter
IBD: infectious bursal disease
NGS: next generation sequencing
SPF: specific-pathogen-free
rRT-PCR: real time RT-PCR
vvIBDV: very virulent infectious bursal disease virus
